# Reply to 'Flawed assumptions compromise water yield assessment'

**DOI:** 10.1038/s41467-018-07065-5

**Published:** 2018-11-15

**Authors:** Ping Zhou, Qiang Li, Guoyi Zhou, Xiaohua Wei, Mingfang Zhang, Zhiyong Liu, Xiuzhi Chen, Xiaodong Liu, Yin Xiao, Ge Sun, David F. Scott, Shuyidan Zhou, Liusheng Han, Yongxian Su

**Affiliations:** 1 Key Lab of Guangdong for Utilization of Remote Sensing and Geographical Information System, Guangzhou Institute of Geography, Guangdong Academy of Sciences, Guangzhou, 510070 China; 2 Department of Earth, Environmental and Geographic Sciences, University of British Columbia, Okanagan, Kelowna, BC V1V1V7 Canada; 3 South China Botanical Garden, Chinese Academy of Sciences, Guangzhou, 510650 China; 4 Center for Information Geoscience, University of Electronic Science and Technology of China, Chengdu, 611731 China; 5 School of Civil Engineering, Sun Yat-sen University, Guangzhou, 510275 China; 6 South China Agricultural University, Guangzhou, 510642 China; 7 Zhengzhou No. 1 Middle School, Zhengzhou, 450000 China; 8 Eastern Forest Environment Threat Assessment Center, USDA Forest Service, Raleigh, NC 27606 USA; 9 Institute of Urban Environment, Chinese Academy of Sciences, Xiamen, 361021 China; 10 Shandong University of Technology, Zibo, 255000 China; 11 Guangzhou Institute of Geography, Guangzhou, 510070 China

## Introduction

We appreciate the comments on the relative contributions of climate and watershed condition to water yield by Gudmundsson et al.^1^ (hereafter G17) on our study^2^ (hereafter Z15), both of which investigated the global pattern of the effect of climate and land cover on water yield. We acknowledge their adoption of a more commonly used definition on the relative contribution. In this correspondence, we confirm the validity of the major conclusions of Z15 and compare the definitions and calculations of the relative contributions between Z15 and G17. We also identify an important research gap on the most appropriate estimation of the relative contributions of climate and land cover to the annual water yield using these global theoretic frameworks (e.g., Fuh^3^ and Budyko), which require further attention.

Z15 first validated the broad application of the Fuh framework (Eqs. 1 and 2) using published watershed data across the globe. Then, Z15 used the theoretical reasoning of the Fuh framework to identify two critical values (*ψ* = *P*/PET = 1 and *m* = 2) and their corresponding hydrological sensitivities and concluded that changes in land cover in watersheds with *ψ* < 1 or *m* < 2 can lead to greater hydrological alterations. In addition, Z15 applied those results to estimate the relative contributions of *ψ* and *m* to *R*/*P*, which pertain to an application aspect of the key conclusions.

G17 questioned the calculation of the relative contributions of Z15. It is critical to stress that such questioning was only on the application aspect of Z15 rather than on their central conclusions. However, the title of G17 has led to significant confusion and a misunderstanding of Z15 as well as of global theoretical frameworks (Fuh, Budyko, etc.) in scientific communities. Thus, we further confirm that the central conclusions of Z15 were not questioned by G17.

G17 also raised a question regarding the physical inconsistency between the results shown in Fig. 7a of Z15 and the central statements presented in the abstract of Z15. The results presented in Fig. 7a of Z15 are about the relative contributions (Eq. (3)), whereas the central statements in the abstract of Z15 refer to sensitivity (Eqs. 10 and 11). Here, we acknowledge that “more responsive” in the abstract of Z15 should be “more sensitive,” and this imprecise wording may lead to G17’s misunderstanding of Fig. 7a of Z15 and the statement in the abstract of Z15.

1 $$\frac{R}{P} = Z\left( {\psi ,m} \right) = \left( {1 + \psi ^{ - m}} \right)^{1/m} - \psi ^{ - 1}{,}$$

or

2 $$\frac{R}{P} = Z\left( {\phi ,m} \right) = \left( {1 + \phi ^m} \right)^{1/m} - \phi{,}$$

where *ψ* is defined as the wetness index ($\psi = P/{\mathrm{PET}}$ in Eq. (1) and *ϕ* is the dryness index ($\phi = {\mathrm{PET}}/P$ in Eq. (2)).

The central debate between Z15 and G17 lies in the relative contributions of *ψ* and *m* to *R*/*P*. The relative contributions can be defined in various ways depending on the research objectives. Z15 defined them as the relative contributions of *ψ* and *m* to the sensitivities of *R*/*P* (Eq. (3)), aiming to compare the relative magnitudes of the sensitivity of *ψ* and *m* to the sensitivities of *R*/*P*. By contrast, G17 adapted a more commonly used definition, as shown in Eq. (5), which can be interpreted as the changed amounts of *R*/*P* due to the changes in *ψ* and *m*. Clearly, two definitions reflect different physical variables of interest, and consequently they are not comparable.

3 $$C_{m,\psi } = \frac{{100}}{{1 + \left| \alpha \right|}},\,{\mathrm{where}} \, \alpha = \left( {\frac{{\partial Z}}{{\partial \psi }}} \right)/\left( {\frac{{\partial Z}}{{\partial m}}} \right){,}$$

4 $$C^ \ast _{m,\phi } = \frac{{100}}{{1 + \left| {\alpha ^ \ast } \right|}},\,{\mathrm{where}} \, \alpha ^ \ast = \left( {\frac{{\partial Z^ \ast }}{{\partial \phi }}} \right)/\left( {\frac{{\partial Z^ \ast }}{{\partial m}}} \right){,}$$

5 $$C_{m,corr} = \frac{{100}}{{1 + \left| \beta \right|}},\,{\mathrm{where}} \, \beta = \left( {\frac{{\partial Z}}{{\partial \psi }}\Delta \psi } \right)/\left( {\frac{{\partial Z}}{{\partial m}}\Delta m} \right){.}$$

**Fig. 1:**
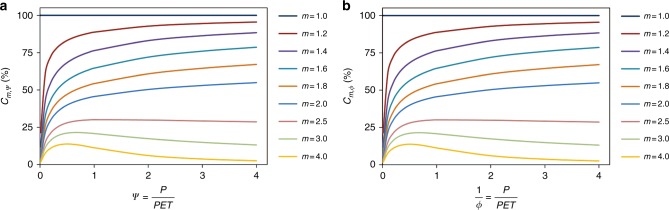
The relative contribution of *m* to changes in *R*/*P* with regard to *P*/PET. **a** *C*_*m,ψ*_ is computed using the wetness index given by Eq. (3) and **b** *C*_*m,ϕ*_ is computed using the dryness index given by Eq. (6)

Using the definition of Z15, G17 further challenged the physical consistency of the approach suggested by Z15 due to the rearrangement of the Fuh framework from *ψ* (*P*/PET) to *ϕ* (PET/*P*) (Eqs. (3) and (4)). As a result, G17 found two different patterns (Fig. 1a, c in G17). We believe that G17 made a simple mistake by assuming *C*_*m*_ (Eq. (3)) and C^*^_m_ (Eq. (4)) to be the same. Although they all refer to the relative contributions of *m*, the former is relative to the climate with regard to wetness (*P*/PET), whereas the latter is relative to the climate with regard to dryness (PET/*P*). Therefore, it is no surprise that they have different trends.

**Fig. 2:**
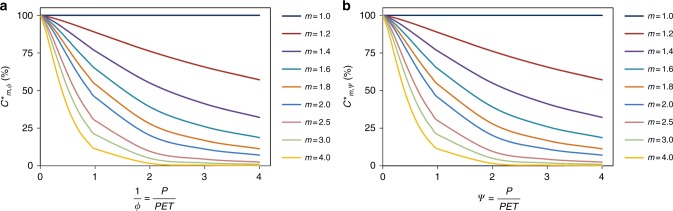
The relative contribution of *m* to changes of *R*/*P* with regard to PET/*P*. **a** C^*^_mϕ_ is computed using the dryness index given by Eq. (4) and **b** C^*^_m,ψ_ is computed using the wetness index given by Eq. (7)

To further demonstrate the physical consistency of Z15, Eqs. (3) and (4) can be rewritten as Eqs. (6) and (7), respectively, when *ϕ* is rearranged to *ψ* (or *ψ* to *ϕ*; see Eqs. (8)–(10) for the derivation in detail). As shown in Figs. 1 and 2, *C*_*m,ψ*_ and *C*_*mϕ*_, are identical in disregard to the rearrangement from *ψ* (*P*/PET) to *ϕ* (PET/*P*). The same phenomenon is also found for C^*^_mϕ_ and C^*^_mψ_. Therefore, Figs. 1 and 2 clearly prove that the approaches in Z15 are physically consistent.

6 $$C_{m,\phi } = \frac{{100}}{{1 + \left| {\frac{{\partial Z}}{{\partial \phi }} \times \frac{{\mathrm{d}\phi }}{{\mathrm{d}\psi }}{\mathrm{/}}\frac{{\partial Z}}{{\partial m}}} \right|}} = \frac{{100}}{{1 + \left| { - \frac{1}{{\psi ^2}} \times \frac{{\partial Z^ \ast }}{{\partial \phi }}{\mathrm{/}}\frac{{\partial Z^ \ast }}{{\partial m}}} \right|}} = \frac{{100}}{{1 + \left| {\phi ^2\alpha ^ \ast } \right|}}{,}$$

7 $$C^ \ast _{m,\psi } = \frac{{100}}{{1 + \left| {\frac{{\partial Z^ \ast }}{{\partial \psi }} \times \frac{{\mathrm{d}\psi }}{{\mathrm{d}\phi }}{\mathrm{/}}\frac{{\partial Z^ \ast }}{{\partial m}}} \right|}} = \frac{{100}}{{1 + \left| { - \frac{1}{{\phi ^2}} \times \frac{{\partial Z}}{{\partial \psi }}{\mathrm{/}}\frac{{\partial Z}}{{\partial m}}} \right|}} = \frac{{100}}{{1 + \left| {\psi ^2\alpha } \right|}}{.}$$

## Methods

### Sensitivities of *R*/*P* to *ψ* (*S*_*ψ*_) and *m* (*S*_*m*_)

The sensitivities of *R*/*P* to *ψ* (*S*_*ψ*_) and *m* (*S*_*m*_) can be derived from Eq. (1) as:

8 $$S_\psi = \frac{{\partial Z}}{{\partial \psi }} = \psi ^{ - 2} - \psi ^{ - m - 1} \times \left( {1 + \psi ^{ - m}} \right)^{\frac{{1 - m}}{m}}{,}$$

9 $$S_m = \frac{{\partial Z}}{{\partial m}} = - \left( {1 + \psi ^{ - m}} \right)^{\frac{1}{m}} \times \left( {\frac{1}{{m^2}} \times \ln \left( {1 + \psi ^{ - m}} \right) + \frac{1}{m} \times \psi ^{ - m} \times \frac{{\ln \psi }}{{1 + \psi ^{ - m}}}} \right){.}$$

### Sensitivities of *R*/*P* to *ϕ* (*S*_*ϕ*_) and *m* (S^*^_m_)

The sensitivities of *R*/*P* to *ϕ* (*S*_*ϕ*_) and *m* (S^*^_m_) can be derived from Eq. (2) as:

10 $$S_\phi = \frac{{\partial Z}}{{\partial \phi }} = \left( {1 + \phi ^m} \right)^{\frac{1}{m}} \times \frac{{\phi ^{m - 1}}}{{1 + \phi ^m}} - 1{,}$$

11 $$S^ \ast _m = \frac{{\partial Z}}{{\partial m}} = \left( {1 + \varphi ^m} \right)^{\frac{1}{m}} \times \left( { - \frac{1}{{m^2}} \times \ln \left( {1 + \varphi ^m} \right) + \frac{1}{m} \times \varphi ^m \times \frac{{\ln \varphi }}{{1 + \varphi ^m}}} \right){.}$$
